# Association of plasma osteoprotegerin and adiponectin with arterial function, cardiac function and metabolism in asymptomatic type 2 diabetic men

**DOI:** 10.1186/1475-2840-10-67

**Published:** 2011-07-19

**Authors:** Weena JY Chen, Luuk J Rijzewijk, Rutger W van der Meer, Martijn W Heymans, Eelco van Duinkerken, Mark Lubberink, Adriaan A Lammertsma, Hildo J Lamb, Albert de Roos, Johannes A Romijn, Jan WA Smit, Jeroen J Bax, Mette Bjerre, Jan Frystyk, Allan Flyvbjerg, Michaela Diamant

**Affiliations:** 1 Diabetes Center/Department of Internal Medicine, VU University Medical Center, Amsterdam, the Netherlands; 2 Department of Radiology, Leiden University Medical Center, Leiden, the Netherlands; 3 Department of Epidemiology and Biostatistics and the EMGO Institute for Health and Care Research, VU University Medical Center, Amsterdam, the Netherlands; 4 Department of Nuclear Medicine & PET Research, VU University Medical Center, Amsterdam, the Netherlands; 5 Department of Endocrinology and Metabolic Diseases, Leiden University Medical Center, Leiden, the Netherlands; 6 Department of Cardiology, Leiden University Medical Center, Leiden, the Netherlands; 7 Medical Department of Endocrinology and Internal Medicine, Aarhus University Hospital & The Medical Research Laboratories, Clinical Institute, Aarhus University, Aarhus, Denmark

**Keywords:** osteoprotegerin, adiponectin, type 2 diabetes mellitus, arterial function, cardiac function, myocardial metabolism

## Abstract

**Background:**

Osteoprotegerin (OPG), a soluble member of the tumor necrosis factor receptor superfamily, is linked to cardiovascular disease. Negative associations exist between circulating OPG and cardiac function. The adipocytokine adiponectin (ADPN) is downregulated in type 2 diabetes mellitus (T2DM) and coronary artery disease and shows an inverse correlation with insulin sensitivity and cardiovascular disease risk. We assessed the relationship of plasma OPG and ADPN and arterial function, cardiac function and myocardial glucose metabolism in T2DM.

**Methods:**

We included 78 asymptomatic men with uncomplicated, well-controlled T2DM, without inducible ischemia, assessed by dobutamine-stress echocardiography, and 14 age-matched controls. Cardiac function was measured by magnetic resonance imaging, myocardial glucose metabolism (MMRglu) by 18F-2-fluoro-2-deoxy-D-glucose positron emission tomography. OPG and ADPN levels were measured in plasma.

**Results:**

T2DM patients vs. controls showed lower aortic distensibility, left ventricular (LV) volumes, impaired LV diastolic function and MMRglu (all P < 0.05). In T2DM men vs. controls, OPG levels were higher (P = 0.02), whereas ADPN concentrations were decreased (P = 0.04). OPG correlated inversely with aortic distensibility, LV volumes and E/A ratio (diastolic function), and positively with LV mass/volume ratio (all P < 0.05). Regression analyses showed the associations with aortic distensibility and LV mass/volume ratio to be independent of age-, blood pressure- and glycated hemoglobin (HbA1c). However, the associations with LV volumes and E/A ratio were dependent of these parameters. ADPN correlated positively with MMRglu (*P *< 0.05), which, in multiple regression analysis, was dependent of whole-body insulin sensitivity, HbA1c and waist.

**Conclusions:**

OPG was inversely associated with aortic distensibility, LV volumes and LV diastolic function, while ADPN was positively associated with MMRglu. These findings indicate that in asymptomatic men with uncomplicated T2DM, OPG and ADPN may be markers of underlying mechanisms linking the diabetic state to cardiac abnormalities.

**Trial registration:**

Current Controlled Trials ISRCTN53177482

## Background

Type 2 diabetes mellitus (T2DM) patients are at high risk of developing cardiovascular disease [1]. Even in the absence of hypertension and coronary artery disease, left ventricular (LV) diastolic dysfunction occurs in a large proportion of asymptomatic T2DM patients [2,3]. Identification of the individual risk is important for the prevention and treatment of cardiovascular disease. Appropriate biomarkers could be important for risk stratification.

Osteoprotegerin (OPG) was first described as a key factor in bone remodeling [4]. OPG is a member of the tumor necrosis factor (TNF) receptor family, and a decoy receptor for the receptor activator of nuclear factor-κB ligand (RANKL) and TNF-α related apoptosis-inducing ligand. It prevents RANKL from binding to its receptor on osteoclasts, thereby inhibiting osteoclastogenesis [4]. Furthermore, OPG has been implicated in human atherogenesis [5,6]. Previously, an association between OPG and LV dysfunction in the general population was described [7]. In addition, elevated OPG levels were present in patients with heart failure [8]. Recently, circulating OPG levels were reported as independent predictor of cardiovascular mortality in patients with stable coronary artery disease [9]. In asymptomatic T2DM patients OPG levels predicted subclinical atherosclerosis and cardiovascular events [10]. A 17-year prospective observational study in T2DM patients showed a strong predictive value of OPG for all-cause mortality, independent of conventional risk for cardiovascular disease, including renal function [11].

The adipocytokine adiponectin (ADPN) is downregulated in T2DM [12]. This hypoadiponectinemia in T2DM is associated with impaired insulin sensitivity, and to a lesser extent with adiposity and glycemia [13]. Furthermore, lower adiponectin levels have been found in T2DM patients with diabetic foot, a complication characterized by neuropathy, microvascular defects and inflammation compared to T2DM patients without diabetic foot [14]. Among men with T2DM, ADPN was negatively associated with the risk of coronary artery disease, which seemed to be partly mediated by high density lipoprotein (HDL)-cholesterol, but independent of other blood lipids, glycated hemoglobin (HbA1c), or inflammatory markers [15].

The present study was based on the hypotheses that circulating OPG and ADPN levels may differ between asymptomatic men with uncomplicated T2DM and age matched normoglycemic controls and that these biomarkers may be associated with arterial function, cardiac function and metabolism in these subjects.

## Methods

### Subjects

This cross-sectional study was a substudy of the previously published Pioglitazone Influence on tRiglyceride Accumulation in the Myocardium in Diabetes (PIRAMID) study [2,16,17]. In short, 78 asymptomatic men with well-controlled, uncomplicated T2DM, in whom inducible ischemia was excluded by dobutamine-stress echocardiography, and 14 age-matched healthy normoglycemic, as ascertained by an oral glucose tolerance test, men were included. All T2DM patients received glimepiride monotherapy. Inclusion criteria were HbA1c 6.5-8.5% at screening, body mass index [BMI; weight/(lenght^2^)] 25-32 kg/m^2 ^and blood pressure not exceeding 150/85 mmHg. Exclusion criteria included known cardiovascular disease, kidney disease, hepatic disease or impaired hepatic function, diabetes related complications, including proliferative retinopathy; microalbuminuria; autonomic neuropathy as assessed by Ewing's tests [18], current or previous use of thiazolidinediones, incretin-based treatments and insulin, and contraindications for magnetic resonance imaging (MRI).

The protocol was approved by the Medical Ethics Review Committee of the VU University Medical Center and Leiden University Medical Center, and the study was performed in full compliance with the Declaration of Helsinki. All participants provided written informed consent prior to inclusion.

### Cardiac MRI protocol

MRI assessments were performed after an overnight fast using a 1.5 Tesla whole-body MRI scanner (Gyroscan ACS/NT15; Philips, Best, the Netherlands), described previously [2,16,17,19]. Images were analyzed quantitatively using dedicated software (MASS and FLOW, Medis, Leiden, the Netherlands).

A retrospective ECG-gated gradient-echo sequence with velocity encoding was applied to measure through plane flow at a predefined position at the mid ascending aorta. Imaging parameters included the following: echo time = 4.83 ms, repetition time = 14 ms, flip-angle = 20°, slice thickness = 8 mm, field of view = 350 mm, matrix size = 256 × 256, velocity encoding gradient (Venc) = 150 cm/s, scan percentage = 80%. The temporal resolution was approximately 25 ms depending on the heart rate. The in-plane spatial resolution was 1.37 × 1.76 mm after reconstruction.

Distensibility of the aorta derived from flow measurements at the mid ascending aorta was calculated using this formula:

Where D = distensibility (mmHg^-1^), A_max _= maximal aortic area (mm^2^), A_min _= minimal aortic area (mm^2^), pulse pressure = systolic blood pressure-diastolic blood pressure (mmHg) [20].

The entire heart was imaged in short-axis orientation using ECG-gated breathholds with a sensitivity encoding balanced turbo-field echo sequence. LV ejection fraction was assessed for the determination of LV systolic function. In addition, LV mass/(enddiastolic) volume ratio was calculated. Imaging parameters included the following: echo time = 6 ms, repetition time = 11 ms, temporal resolution = 35-39 ms per cardiac phase, depending on the heart rate, flip-angle = 30°, slice thickness = 10 mm, field of view = 400 mm, matrix size = 256 × 256. Furthermore, an ECG-gated gradient-echo sequence with velocity encoding was performed to measure blood flow across the mitral valve for determination of LV diastolic parameters. Imaging parameters included the following: echo time = 4.83 ms, repetition time = 14 ms, flip-angle = 20°, slice thickness = 8 mm, field of view = 350 mm, matrix size = 256 × 256, Venc = 100 cm/s, scan percentage = 80%. The resulting biphasic diastolic inflow pattern consists of 2 peaks, representing the early filling phase and the atrial contraction. Analysis of the early filling phase and the atrial contraction was performed by calculating their peak filling rates and ratio of the peak filling rates (E/A). In addition, the peak deceleration gradient of the early filling phase (E deceleration peak) was calculated automatically. An estimation of LV filling pressures (E/Ea) was assessed [21]. During MRI, blood pressure and heart rate were measured.

### Positron Emission Tomography (PET) protocol

PET assessments were performed after an overnight fast using an ECAT EXACT HR+ scanner (Siemens/CTI, Knoxville, TN, USA). Myocardial blood flow was measured with H_2_^15^O in the fasting state. After a 10-minutes transmission scan, 1100 MBq H_2_^15^O was injected, and a dynamic emission scan (40 frames) was acquired. Myocardial metabolic rate of glucose (MMRglu) was measured during an euglycemic-clamp procedure [22] using [^18^F]-2-fluoro-2-deoxy-D-glucose (^18^FDG). A 60-minutes dynamic emission scan (40 frames) was acquired following injection of 185 MBq^18^FDG [17].

### PET Image Analysis

PET data were quantitatively reconstructed with filtered backprojection applying all appropriate corrections. To generate myocardial time-activity curves, regions of interest were defined on resliced LV short-axis (summed)^18^FDG images and subsequently projected onto the dynamic images. The regions of interest were drawn as previously described [23] and grouped for further analysis. Additional regions of interest were defined in left and right ventricular chambers for H_2_^15^O image-derived input functions. A separate aorta ascendence region of interest was defined for^18^FDG image-derived input functions. Myocardial blood flow was determined with the standard single-tissue compartment model [24]. MMRglu was calculated by multiplying the net influx constant for^18^FDG, *K*_i_, by the mean plasma glucose concentration. For determining of *K*_i_, Patlak graphic analysis was used [25].

### Biochemical analyses

Fasting plasma OPG was measured using a sandwich enzyme-linked immunosorbent assay and commercially available antibodies (R&D Systems, Minneapolis, MN, USA). The range of the assay was 62.5-4000 ng/L. The intra-assay coefficient of variation was 3.5% [26]. Fasting plasma ADPN was measured using a time-resolved immunofluorometric assay based on two monoclonal antibodies and recombinant human adiponectin (R&D Systems, Abingdon, UK). The intra-assay coefficient of variation was <5%, and the interassay coefficient of variation was <10% [26,27]. N-terminal-pro-B-type natriuretic peptide (NT-pro-BNP) was measured using an electrochemiluminescence immunoassay (Roche Diagnostics GmbH, Mannheim, Germany). The intra-assay coefficient of variation was 1.5%, and the inter-assay coefficient of variation was 1.9%.

### Statistical analysis

Data are expressed as means ± standard deviations for normally distributed data, or otherwise the median (interquartile range) is used. T-tests or Mann-Whitney U tests were used to determine group differences. Correlation coefficients were calculated using the Pearson's product moment correlation. OPG and ADPN were log-transformed for correlation and multivariable regression analyses as both markers were non-normally distributed. Linearity of the regression models was judged based on histograms and scatterplots. As no interactions were found between group and dependent variables, both groups were analyzed as one for regression analysis. Additional potential confounders were investigated by adding age, group status, HbA1c, NT-pro-BNP, and blood pressure for associations of OPG and age, group status, HDL-cholesterol, whole-body insulin sensitivity (M-value), HbA1c, BMI, and waist for association of ADPN one by one to the crude models. Variables that changed the regression coefficients by more than 10% were included in the adjusted model. Statistical analyses were performed using SPSS Software (version 15.0, SPSS Inc., Chicago, IL, USA). Values of P < 0.05 were considered statistically significant.

## Results

As expected, T2DM patients versus healthy men had increased BMI, waist, and HbA1c values, but lower HDL-cholesterol, and M-value. Although within the normal range, systolic and diastolic blood pressure and heart rate were significantly higher in T2DM patients relative to controls (Table 1). T2DM patients had a lower aortic distensibility as compared to controls. Furthermore, T2DM patients and controls had comparable LV mass and systolic function parameters. Parameters related to LV volume and diastolic function were, however, significantly decreased in patients (Table 2). Insulin stimulated MMRglu was significantly impaired in patients compared with controls, in the presence of comparable myocardial blood flow [2,17] (Table 2).

**Table 1:** Clinical and biochemical characteristics of study subjects

	T2DM (n = 78)	Controls (n = 14)	P value
Age (years)	56.5 ± 5.6*	54.5 ± 7.1	0.14
BMI (kg/m^2^)	28.7 ± 3.5*	27.4 ± 2.3	0.04
Waist (cm)	104.4 ± 10.2*	99.2 ± 8.9	0.03
Heart rate (beats/min)	61.7 ± 8.6	54.0 ± 7.8	0.004
Systolic blood pressure (mmHg)	130 ± 12*	121 ± 8	0.002
Diastolic blood pressure (mmHg)	82 ± 8 *	74 ± 7	< 0.001
Antihypertensive medication (%)	44*	NA	
Lipid lowering medication (%)	49*	NA	
Fasting plasma glucose (mmol/L)	8.3 (7.1-10.0)*	5.3 (5.1-5.5)	< 0.001
Fasting insulin (pmol/L)	64 (36-92)*	29 (19-32)	0.001
HbA1c (%)	7.1 ± 1.0*	5.3 ± 0.2	< 0.001
Total cholesterol (mmol/L)	4.7 ± 1.0*	5.3 ± 0.7	0.01
HDL-cholesterol (mmol/L)	1.1 (0.9-1.3)*	1.4 (1.3-1.6)	< 0.001
NT-proBNP (ng/L)	27 (20-42)*	28 (21-76)	0.26
M-Value (mg/kg min)	2.7 (1.6-4.2)	6.7 (4.3-8.1)	< 0.001

*Adapted from Rijzewijk *et al*. [2]. Data are represented as means ± SD or as median (interquartile range). BMI,: body mass index; NA: not applicable; HbA1c: glycated hemoglobin; HDL: high-density lipoprotein; M-value: whole-body insulin sensitivity; NT-pro-BNP: N-terminal-pro-B-type natriuretic peptide.

**Table 2:** Cardiac dimensions, function and glucose metabolism

	T2DM (n = 78)	Controls (n = 14)	P value
Rate pressure product ((beats/min)mmHg)	8503 ± 1458	7096 ± 1531	0.005
Distensibility aorta ascendens (10^-3^mmHg^-1^)	3.13 (2.29-5.95)	5.63 (4.50-8.07)	0.008
LV mass (g)	107.3 ± 16.7*	111.7 ± 24.4	0.42
LV enddiastolic volume (mL)	154 (134-174)	180 (159-210)	0.002
LV end-systolic volume (mL)	59 (52-71)*	72 (63-82)	0.001
LV mass/volume ratio (g/mL)	0.70 ± 0.11	0.63 ± 0.09	0.05
Stroke volume (mL)	94 ± 16*	107 ± 23	0.002
LV ejection fraction (%)	60 ± 6*	59 ± 4	0.35
E peak filling rate (mL/s)	417 ± 84*	503 ± 112	< 0.001
E deceleration peak (mL/s^2 ^10^-3^)	3.40 (2.89-3.99)*	4.73 (3.11-5.19)	0.003
E/A peak ratio	1.04 ± 0.25*	1.26 ± 0.36	0.01
E/Ea	9.3 (7.0-11.7)	8.7 (6.4-10.8)	0.34
MBF (mL/g/min)	0.84 (0.75-0.96)	0.88 (0.77-1.01)	0.8
MMRglu (nmol/mL/min)	257 ± 130	348 ± 154	0.02

*Adapted from Rijzewijk *et al*. [2]. Data are represented as means ± SD or as median (interquartile range). LV: left ventricular; E/A: ratio of peak filling rates of the early filling phase and arterial contraction; E/Ea: estimated LV filling pressures; MBF: myocardial blood flow; MMRglu: myocardial metabolic rate of glucose uptake.

**Figure 1:**
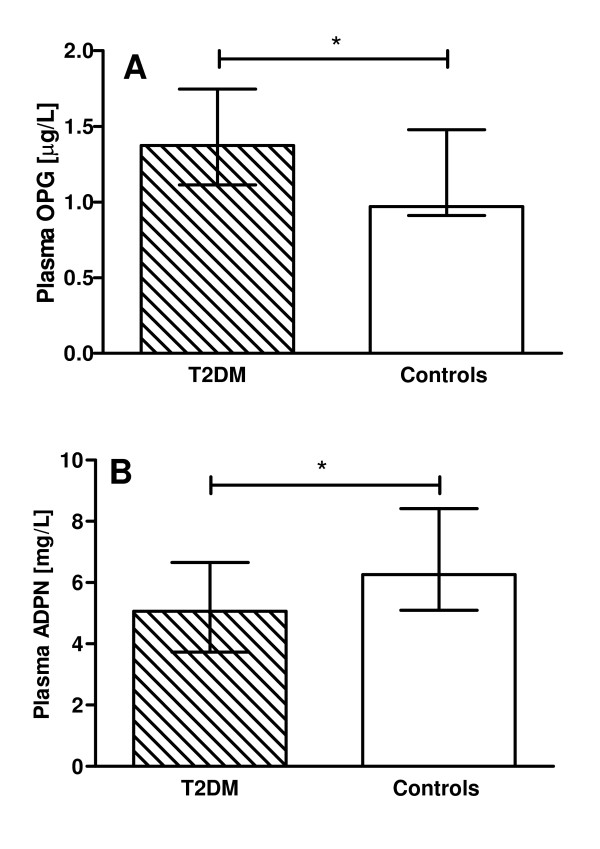
**Plasma levels of osteoprotegerin (OPG) and adiponectin (ADPN) of type 2 diabetic (T2DM) men (hatched bar) and normoglycemic healthy controls (white bar)**. Bars represent median (interquartile range). * P < 0.05.

**Figure 2:**
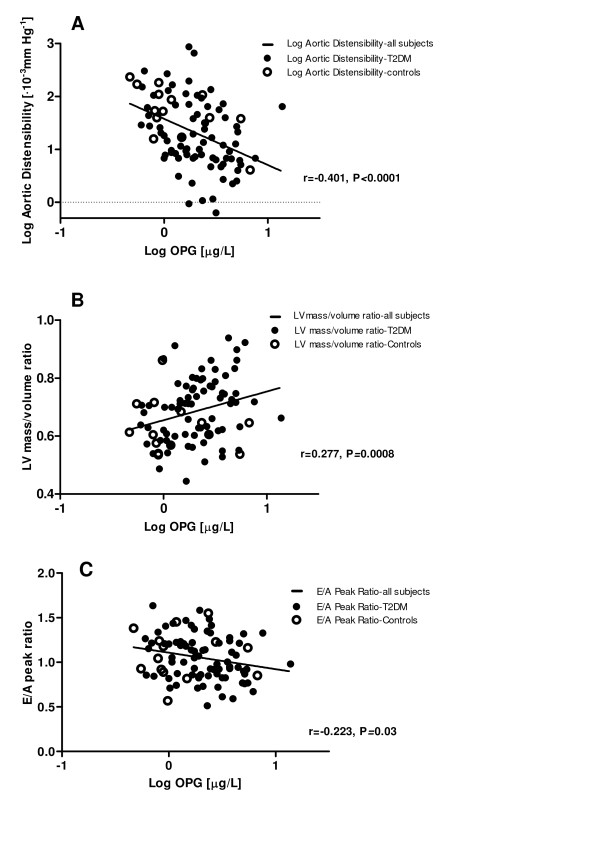
**Scatterplots with fitted regression curves (unadjusted) of Log osteoprotegerin (OPG) and Log aortic distensibility, Log OPG and left ventricular (LV) mass/volume ratio, and Log OPG and E/A peak ratio, for all subjects**.

Plasma OPG levels were significantly elevated in T2DM men compared to controls (Figure 1A), whereas ADPN levels were lower (Figure 1B). PET examinations were successful in 58 T2DM patients and 9 controls. In T2DM patients, plasma OPG levels correlated inversely with aortic distensibility, LV endsystolic volume, E/A peak ratio and MMRglu, and positively with age, and HbA1c and LV mass/volume ratio. The correlations of plasma OPG with systolic blood pressure and LV enddiastolic volume were only significant in pooled analyses (Table 3). In multivariable regression analysis, plasma OPG was negatively associated with aortic distensibility, even after adjustment for systolic blood pressure and age (β = -0.268, P = 0.007; Figure 2A). Likewise, the association of plasma OPG and LV mass/volume ratio was independent of systolic blood pressure and age (β = 0.216, P = 0.05; Figure 2B). However, age and blood pressure significantly affected the association between OPG and LV enddiastolic volume in the T2DM patients (β = -0.104, P = 0.32). In addition, age and HbA1c were contributors of the association between OPG and E/A peak ratio (β = -0.071, P = 0.47; Figure 2C).

**Table 3:** Correlations between osteoprotegerin and clinical and biochemical characteristics, and cardiac dimensions, function, and myocardial metabolism

	Pooled analysis	T2DM	Controls
Age (years)	0.217*	0.343*	-0.354
BMI (kg/m^2^)	0.054	0.050	-0.180
Systolic blood pressure (mmHg)	0.232*	0.181	0.297
Diastolic blood pressure (mmHg)	0.194	0.180	0.192
HbA1c (%)	0.325*	0.249*	0.312
Rate pressure product ((beats/min)mmHg)	0.200	0.114	0.319
Log distensibility aorta ascendens (10^-3^mm Hg^-1^)	-0.401*	-0.327*	-0.623*
LV mass (g)	0.034	0.096	-0.180
LV mass/volume ratio (g/mL)	0.277*	0.311*	-0.164
Log LV enddiastolic volume (mL)	-0.213*	-0.211	-0.044
Log LV endsystolic volume (mL)	-0.269*	-0.248*	-0.116
LV ejection fraction (%)	0.199	0.161	0.343
E peak filling rate (mL/s)	-0.190	-0.193	-0.022
Log E deceleration peak (mL/s^2^10^-3^)	-0.167	-0.156	-0.083
E/A peak ratio	-0.223*	-0.272*	-0.095
MMRglu (nmol/mL/min)	-0.308*	-0.307*	-0.055

Data are Pearson's r. BMI: body mass index; HbA1c: glycated hemoglobin; LV: left ventricular; E/A: ratio of peak filling rates of the early filling phase and arterial contraction; MMRglu: myocardial metabolic rate of glucose uptake.

* P < 0.05.

**Figure 3:**
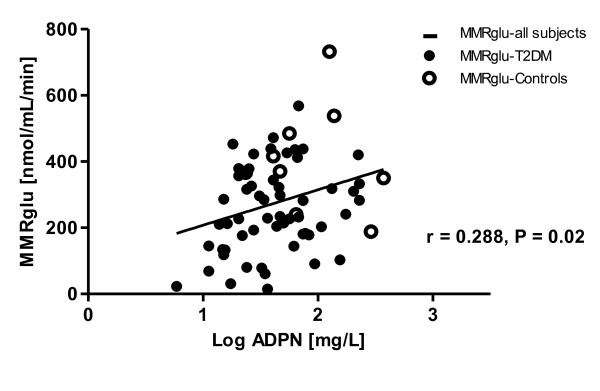
**Scatterplot with fitted regression curves (unadjusted) of Log adiponectin (ADPN) and myocardial metabolic rate of glucose uptake (MMRglu) for all subjects**.

Plasma ADPN levels were inversely correlated with BMI and waist, and positively with HDL-cholesterol and M-value, in T2DM patients. Correlations of ADPN and HbA1c, rate pressure product and MMRglu were only significant in pooled analyses. ADPN was not significantly correlated with LV functional parameters (Table 4). In multiple regression analysis, M-value, HbA1c and waist were contributors of the association between ADPN and MMRglu (β = 0.055, P = 0.66; Figure 3).

**Table 4:** Correlations between adiponectin and clinical and biochemical characteristics, and cardiac dimensions, function, and myocardial metabolism

	Pooled analysis	T2DM	Controls
Age (years)	-0.070	-0.054	0.037
BMI (kg/m^2^)	-0.224*	-0.299*	0.495
Waist (cm)	-0.284*	-0.314*	0.153
Systolic blood pressure (mmHg)	-0.123	-0.070	0.644
Diastolic blood pressure (mmHg)	0.038	0.180	0.083
HbA1c (%)	-0.253*	-0.095	-0.616*
Log HDL-cholesterol (mmol/liter)	0.414*	0.397*	0.192
Log M-Value (mg/kg min)	0.514*	0.485*	-0.130
Rate pressure product ((beats/min)mmHg)	-0.364*	-0.270	-0.537
LV mass (g)	-0.015	-0.004	0.083
LV ejection fraction (%)	0.051	0.112	-0.338
E peak filling rate (mL/s)	0.146	0.082	0.179
Log E deceleration peak (mL/s^2^10^-3^)	0.091	0.073	-0.035
E/A peak ratio	-0.027	-0.054	-0.018
MMRglu (nmol/mL/min)	0.288*	0.251	-0.066

Data are Pearson's r. BMI: body mass index; HbA1c: glycated hemoglobin; HDL: high-density lipoprotein; M-value: whole-body insulin sensitivity; LV: left ventricular; E/A: ratio of peak filling rates of the early filling phase and arterial contraction; MMRglu: myocardial metabolic rate of glucose uptake.

* P < 0.05.

## Discussion

The present study confirmed the previously reported increase in circulating OPG and decrease in ADPN in patients with T2DM versus health controls. We extended these data by showing inverse associations of OPG and cardiac function as well as aortic distensibility, whereas ADPN was positively related to cardiac metabolism.

These correlations were seen both in pooled analyses and the analyses of T2DM men separately, but not in the analyses of the controls alone.

Higher levels of OPG have documented in T2DM patients with asymptomatic coronary artery disease [28,29], but at the same time also in experimental and clinical heart failure, even in the absence of ischemic cardiomyopathy. Similarly, in our population inducible ischemia was excluded. OPG has been associated with progression of atherosclerosis in symptomatic cardiovascular disease. For example, in a population based cohort, serum OPG was significantly associated with myocardial infarction, ischemic stroke, and total mortality [30]. Although, some indicate a beneficial role of OPG [8,31], the higher levels of OPG have been interpreted as a counter regulatory protective response to atherosclerosis. For instance, selective deletion of OPG in mice results in significant medial calcification of the aorta and renal arteries [31]. Futhermore, Ueland et al. [8] showed enhanced systemic expression of RANKL and increased expression of the receptor RANK in cardiomyocytes, vascular smooth muscle cells and endothelial cells of the failing myocardium. OPG can act as a decoy receptor, binding to RANKL, preventing the interaction of RANK and RANKL. Thereby, OPG can potentially protect against the negative effects of RANKL. Besides, in patients with subclinical atherosclerosis serum OPG levels decreased linearly with increased calcification expressed as an increased echogenicity of the atherosclerotic plaques [32]. In addition to LV diastolic function, in our study, plasma levels OPG were significantly associated with age and HbA1c in T2DM patients, showing close correlation between OPG and several cardiovascular risk factors, as demonstrated previously [10,33]. This is the first study showing an inverse association between OPG levels and arterial- and LV diastolic function in asymptomatic T2DM patients in the absence of inducible myocardial ischemia. The multivariate analysis indicated that plasma OPG may contribute to the effect of aging and hyperglycemia on arterial- and LV diastolic function in T2DM. These results indicate a positive relationship between OPG and cardiovascular disease, instead of a protective role of OPG in cardiovascular disease.

The association between ADPN and systemic glucose metabolism has been documented before by others [12,13]. In the present study, we found a positive association of ADPN and myocardial glucose metabolism, but not with cardiac function. This finding seems to be at odds with previous data, which have described an association of circulating levels of ADPN with parameters of cardiac structure and function in healthy adults [34], elderly [35] and patients with coronary artery disease and/or heart failure [36,37]. In addition, in healthy subjects, circulating ADPN levels were inversely related to LV wall thickness and mass [34] and, in patients with coronary artery disease and/or heart failure, they were positively related to NT-proBNP and LV systolic dysfunction [36,37]. In chronic heart failure, the paradoxical (positive) association of ADPN and cardiac function was ascribed to its role in wasting in cardiac cachexia [38]. Differences in study population, and in methodology used to assess cardiac function and ADPN analysis, respectively, all could have accounted for the differential findings. Indeed, we studied men with well-controlled uncomplicated T2DM and with stress-echocardiography confirmed absence of inducible ischemia, whereas others included healthy adults [34], elderly [35] and patients with coronary artery disease and/or heart failure [36,37].

Although in line with previous investigations, we have determined total circulating ADPN levels in our study, but other data suggest that high-molecular weight ADPN may have stronger associations with insulin resistance and coronary heart disease in T2DM patients [39].

Limitations of this study include the cross-sectional design, which precludes conferring a causal relationship between OPG, ADPN and the cardiac variables studied. Furthermore, the correlations of OPG with cardiac function and ADPN with myocardial glucose uptake were not observed in the healthy controls alone. This may be due to the relatively small number of a homogeneous control population.

In addition, the inclusion of only males with uncomplicated T2DM limits generalizations. On the other hand, a strength of this investigation is the use of state-of-the-art cardiac phenotyping methods in a relatively large group of well-characterized T2DM patients.

## Conclusions

The findings of this study indicate that in asymptomatic men with uncomplicated T2DM, but without inducible ischemia, OPG and ADPN may be markers of underlying mechanisms linking the diabetic state to cardiac abnormalities. The use of OPG and ADPN as markers might add to the risk stratification of (future) cardiac disease in asymptomatic men with less-advanced T2DM prior to the onset of coronary artery disease.

## List of abbreviations

T2DM: type 2 diabetes mellitus; LV: left ventricular; TNF: tumor necrosis factor; RANKL: receptor activator of nuclear factor-κB ligand; ADPN: adiponectin; NT-pro-BNP: N-terminal-pro-B-type natriuretic peptide; HDL: high density lipoprotein; HbA1c: glycated hemoglobin; M-value: whole-body insulin sensitivity; BMI: body mass index; MRI: magnetic resonance imaging; E/A: ratio of peak filling rates of the early filling phase and arterial contraction; E deceleration peak: peak deceleration gradient of the early filling phase; E/Ea: estimated LV filling pressures; PET: Positron Emission Tomography; MMRglu: myocardial metabolic rate of glucose.

## Competing interests

JJB received grants from Medtronic, Boston Scientific, Biotronik, St. Jude Medical, BMS medical imaging, Edwards Lifesciences and GE Healthcare. MD is a consultant and speaker for Eli Lilly and Company, Novo Nordisk and Merck, Sharp and Dohme, and a consultant for Sanofi-Aventis, Astra-Zeneca/BMS and Novartis Pharma. Through M.D. the VU University Medical Center in Amsterdam has received research grants from Amylin Pharmaceuticals Inc, Eli Lilly and Company, Novo Nordisk, Merck, Sharp and Dohme, Novartis and Takeda. The other authors declare that they have no competing interests.

## Authors' contributions

WJYC interpreted the data, performed the statistical analysis and drafted the manuscript. LJR participated in acquisition of the data, coordinated the study, interpretation of the data and critically reviewed the manuscript. RWM participated in acquisition of the data, coordinated the study, interpretation of the data and critically reviewed the manuscript. MWH participated in statistical analysis, interpretation of the data, and critically reviewed the manuscript. ED participated in statistical analysis, interpretation of the data and critically reviewed the manuscript. ML participated in analysis and interpretation of the data, and critically reviewed the manuscript. AAL participated in analysis and interpretation of the data, and critically reviewed the manuscript. HJL contributed to the design of the study, interpretation of the data, and critically reviewed the manuscript. AR participated in analysis and interpretation of the data, and critically reviewed the manuscript. JAR contributed to the design of the study, interpretation of the data, and critically reviewed the manuscript. JWAS contributed to the design of the study, interpretation of the data, and critically reviewed the manuscript. JJB participated in the interpretation of the data, and critically reviewed the manuscript. MB contributed to the interpretation of the data, and critically reviewed the manuscript. JF contributed to the interpretation of the data, and critically reviewed the manuscript. AF contributed to the acquisition of the data, participated in analysis and interpretation of the data and critically reviewed the manuscript. MD contributed to the design of the study, interpretation of the data, and critically reviewed the manuscript. All authors read and approved the final manuscript.
